# Tribbles 3 deficiency promotes atherosclerotic fibrous cap thickening and macrophage-mediated extracellular matrix remodelling

**DOI:** 10.3389/fcvm.2022.948461

**Published:** 2022-08-26

**Authors:** Laura Martinez-Campesino, Klaudia Kocsy, Jaime Cañedo, Jessica M. Johnston, Charlotte E. Moss, Simon A. Johnston, Stephen Hamby, Alison H. Goodall, Jessica Redgrave, Sheila E. Francis, Endre Kiss-Toth, Heather L. Wilson

**Affiliations:** ^1^Department of Infection, Immunity and Cardiovascular Disease, Medical School, University of Sheffield, Sheffield, United Kingdom; ^2^Department of Cardiovascular Sciences, University of Leicester, Leicester, United Kingdom; ^3^National Institute for Healthcare Research, Leicester Biomedical Research Centre, Glenfield Hospital, Leicester, United Kingdom; ^4^Biological Research Centre of the Hungarian Academy of Sciences, Szeged, Hungary

**Keywords:** atherosclerosis, TRIB3, macrophage, fibrous, cap

## Abstract

Tribbles 3 (TRIB3) modulates lipid and glucose metabolism, macrophage lipid uptake, with a gain-of-function variant associated with increased cardiovascular risk. Here we set out to examine the role of this pseudokinase in atherosclerotic plaque development. Human endarterectomy atherosclerotic tissue specimens analysed by immunofluorescence showed upregulated TRIB3 in unstable plaques and an enrichment in unstable regions of stable plaques. Atherosclerosis was induced in full body *Trib3^KO^* and *Trib3^WT^* littermate mice by injecting mPCSK9 expressing adeno-associated virus and western diet feeding for 12 weeks. *Trib3^KO^* mice showed expanded visceral adipose depot while circulatory lipid levels remained unaltered compared to wildtype mice. *Trib3^KO^* mice aortae showed a reduced plaque development and improved plaque stability, with increased fibrous cap thickness and collagen content, which was accompanied by increased macrophage content. Analysis of both mouse and human macrophages with reduced TRIB3 expression showed elongated morphology, increased actin expression and altered regulation of genes involved in extracellular matrix remodelling. In summary, TRIB3 controls plaque development and may be atherogenic *in vivo*. Loss of TRIB3 increases fibrous cap thickness *via* altered metalloproteinase expression in macrophages, thus inhibiting collagen and elastic fibre degradation, suggesting a role for TRIB3 in the formation of unstable plaques.

## Introduction

Atherosclerosis is a progressive disease of the arteries where LDL particles from the circulation accumulate in the arterial intima and undergo modification by oxidation promoting endothelial dysfunction and immune cell recruitment (1, 2). As atherosclerotic lesions progress, the risk of plaque rupture and subsequent thrombosis increasingly depends on plaque composition. Fibrous cap thickness, collagen content, necrotic core size and the inflammatory microenvironment together determine plaque vulnerability, rather than plaque size *per se* (3). Macrophages are key effector cells in the initiation and progression of the atherosclerotic plaque; local signals influence polarisation of this immune cell type toward pro-atherogenic or at hero-protective phenotypes, thereby influencing plaque stability (4). Macrophages in the plaque engulf oxidised lipids and transform into foam cells (5). Further accumulation of foamy macrophages, inflammatory cells, and migration of vascular smooth muscle cells (VSMC) promotes atheroma development. Fibrous cap thickness can be further modulated by activated macrophages, key sources of extracellular matrix metalloproteinases in human and murine atherosclerotic plaques (6, 7).

The pseudokinase Tribbles 3 (TRIB3) has been implicated in a number of metabolic processes such as modulating lipid and glucose metabolism (8, 9), promoting insulin resistance (10–12), and inhibiting adipocyte differentiation (13–15). Human TRIB3 is ubiquitously expressed, with high levels in bone marrow, thyroid gland and peripheral blood leukocytes (16). The Q84R variant of the human TRIB3 gene has been associated with insulin resistance, altered insulin secretion and with increased cardiovascular risk (17) but the molecular mechanisms behind these associations are poorly understood. In a model of diabetic atherosclerosis using *ApoE/Ldldr* double knockout mice fed with high fat and high sugar diet, siRNA-depletion of *Trib3* led to a reduction in insulin resistance and atherosclerotic burden (18). However, the diabetogenic diet used for the development of diabetes-accelerated atherosclerosis prevents a distinction between the systemic effects produced by promoting hyperglycaemia and diabetes from the effects associated with hyperlipidaemia and its local impact on atherosclerosis (19). *In vitro*, using the human THP-1 monocytic cell line, ox-LDL was found to enhance TRIB3 activity; ox-LDL treatment combined with TRIB3 over-expression promotes cholesterol accumulation and decreased production and secretion of pro- inflammatory cytokines IL-1β, MCP-1, and TNF (20).

Here we sought to examine the consequences of altered TRIB3 levels in atherosclerotic plaque development and the molecular mechanisms underpinning these, with a focus on macrophages, using a range of *in vivo* and *in vitro* human and murine models. We show TRIB3 expression is increased in unstable regions of human carotid artery plaques. Loss of TRIB3 in a full body knockout mouse model of atherosclerosis results in reduced atherosclerotic burden *via* increased collagen and fibrous cap thickness and increased macrophage plaque content. Human and mouse macrophages with reduced TRIB3 have altered expression of extracellular matrix metalloproteinases and actin remodelling pathways, resulting in increased actin levels, cellular size, and elongation.

## Materials and methods

### Human carotid plaque isolation and characterisation

Human tissue and blood samples from volunteers and patients who gave informed written consent, were collected under protocols approved by the University of Sheffield Research Ethics Committee and Sheffield Teaching Hospitals Trust Review Board (ref. STH18222 and SMBRER310) and in accordance with the Declaration of Helsinki.

Carotid plaques were removed during carotid endarterectomy and placed in 10% (v/v) neutral buffered formalin following decalcification by placing them in 0.5 M EDTA at pH8 for 7 days. Plaques were then divided and embedded into four 3 mm thick wax blocks (A, B, C, D) centred from the carotid bifurcation. 5 μm tissue sections were obtained from wax blocks using a Leica RM2245 semi-automated rotary microtome and placed on APES microscope slides. Tissue sections were stained with haematoxylin and Eosin (H&E) for histological classification. For immunofluorescence staining against TRIB3 rabbit anti-human TRIB3 ab64693 or ab50516 was used at 1:50 with NL557 anti-rabbit secondary (NL004) or Alexa 488 anti-rabbit secondary (4412S, Cell Signalling) at 1:1,000. Mouse anti-human CD68 (ab201340) was used at 1:50 with Alexa 594 anti-mouse secondary (8890S, Cell Signaling) at 1:1,000. One carotid plaque lesion was mounted per slide and imaged by tile scanning using a Leica AF6000LX inverted microscope with a 5x/0.12 dry objective. The plaque shoulder region was captured with 10x/0.3 or 20x/0.35 dry lens. Regions of interest were analysed across two sections with Fiji. The TRIB3 + area was normalised to the plaque area. The proportion of double positive stained CD68+/TRIB3+ macrophages was compared to the total CD68 + macrophages assessed separately in each fluorescent channel.

### Human monocyte derived macrophage isolation and culture

Human blood samples drawn by venepuncture were mixed with 3.8% (w/v) trisodium citrate dehydrate (Na_3_C_6_O_7_ x H_2_O, Sigma). After gradient centrifugation using Ficoll-Paque PLUS (GE Healthcare), peripheral blood mononuclear cells (PBMCs) were isolated in phosphate buffer saline (PBS) containing 2 mM EDTA. RBC were lysed using an ammonium chloride solution (155 mM NH_4_Cl, 10 mM KHCO_3_, 0.1 M EDTA in H_2_O). Magnetic CD14 microbeads (Miltenyi Biotec) were used for the positive selection of human monocytes. Monocytes were incubated for 7 days at 37^°^C and 5% CO2 in RPMI 1640 medium (Gibco) containing: 10% (v/v) ultra-low endotoxin heat-inactivated foetal bovine serum (FBS) (Biowest); 1% (v/v) L-Glutamine (Lonza); 1% (v/v) penicillin-streptomycin (Gibco); and 100 nM of macrophage colony stimulated factor (M-CSF) (Peprotech). Small-interfering RNAs were transfected into human monocyte derived macrophages (HMDM) to achieve RNA silencing. The transfection reagent Viromer Green (Lipocalyx) was used following the manufacturer’s instructions. siRNA targeting TRIB3 gene (ON-TARGET plus smartpool siRNA #L- 003754-00, Dharmacon) was mixed at 12.5 nM with the transfection reagent. As a control, ON- TARGETplus smartpool control non-targeting siRNA (#D-001810-10-20, Dharmacon) was used at the same concentration.

### Microarray analysis of human monocyte derived macrophages

The published Cardiogenic Consortium transcriptomic data set (21–23) was re-analysed in collaboration with Professor Alison Goodall and Dr Stephen Hamby (University of Leicester, United Kingdom) as described in Johnston et al. (24). The dataset contains microarray analysis of human monocyte derived macrophages from 596 donors (Illumina’s Human Ref-8 Sentrix Bead Chip arrays, Illumina Inc., San Diego, CA). The samples were ranked based on TRIB3 expression and top and bottom quartiles were compared, obtaining 5,782 differentially expressed genes co-expressed with TRIB3. The genes passing the cut off *p*-value < 0.05 and passing threshold fold change < 0.8 (directly correlated) and > 1.2 (indirectly correlated) were further analysed for a functional gene set enrichment analysis using the DAVID Functional Annotation Analysis Software^1^ and Metascape.org (25).

### Experimental animal procedures and atherosclerosis development

All experiments were performed in accordance with UK legislation under the Animals (Scientific Procedures) Act 1986. The University of Sheffield Project Review Committee approved all animal experiments which were carried out under the UK Home Office Project Licence P5395C858. A *Trib3* knock out (*Trib3^KO^*) mouse strain was developed using the gene-trap system targeting the first intron, as described previously (26). The vector contained two expression cassettes: one encoding the splice acceptor site, β-Galactosidase and neomycin; the second encoding a diagnostic marker and splice donor site. Heterozygous mice were backcrossed with C57/BL6 mice for 10 generations. New heterozygous animals were inter-crossed to obtain final *Trib3^KO^* embryos.

Mice were kept in an optimal and controlled environment to reduce stress. Mice were subjected to 12 h light/12 h dark cycle, at 22^°^C with 40–60% of humidity. 12-week-old *Trib3^KO^* and *Trib3^WT^* male mice [*N* = 6 per group, based on power calculations for number of animals from a previous similar study type (24)] were subjected to tail vein injection with an adeno-associated virus-based vector (rAAV8) carrying the mPCSK9-D377Y gene, purchased from UNC GTC Vector Core (Chapel Hill, NC). The mice received 6.1 × 10^11^ viral particles *via* a single injection. Following 7 days of recovery, the mice were fed with Western Rodent Diet (Special Diet Services, United Kingdom), with ingredients [% (w/w)]: Sucrose, 33.94; milk fat anhydrous, 20.00; casein, 19.50; maltodextrin, 10.00; corn starch, 5.00; cellulose, 5.00; corn oil, 1.00; calcium carbonate, 0.40; L-cystine, 0.30; choline bitartrate, 0.20; Cholesterol, 0.15; antioxidant, 0.01; AIN-76A-MX, 3.50; AIN-76A-VX, 1.00. Specification: Crude Fat, 21.4; Crude Protein, 17.5; Crude Fibre, 3.5; Ash, 4.1; Carbohydrate, 50.0.) The diet was fed for 12 weeks, as we described previously (24); atherosclerosis does not develop in the absence of PCSK9 or western diet (27, 28). Weekly weight measurements were performed over the duration of the diet. Mice were culled *via* pharmacological overdose of 0.2 ml sodium pentobarbital (200 mg/ml) applied into the peritoneal cavity followed by cervical dislocation. For mice used to assess atherosclerotic lesions or when blood was required, cervical dislocation was avoided, and cardiac puncture was performed following pentobarbital injection and prior to breath cessation. Following loss of pedal reflex, blood was collected into a heparinised syringe injected to the heart through the chest wall.

### Plasma lipid profiling

Plasma was collected from the mice 3 h after fasting, separated from isolated blood by centrifugation (1,500 × *g* for 5 min at room temperature) and immediately stored at −80^°^C prior to a full lipid profile analysis: total cholesterol, low (LDL), and high (HDL) density lipoproteins, triglycerides and glucose. This was carried out in the Department of Clinical Chemistry at the Royal Hallamshire Hospital (Sheffield Teaching Hospitals) using a Roche Cobas 8000 modular analyser.

### En face staining of the aorta

Quantification of atherosclerosis was performed by *en face* aorta and aortic root staining. Mice were euthanised and the aortic tree was perfused through the heart with PBS and then with 10% (w/v) neutral buffered formalin. Under a dissecting microscope, the aorta was dissected out from the diaphragm to the top of the aortic arch. Following careful cleaning of the adventitia and removal of surrounding material (fat and connective tissue), it was excised longitudinally and fixed in 4% (w/v) paraformaldehyde. Dissected aortas were stained with Oil Red O [ORO, 60% (v/v) in isopropanol] and secured with insect pins onto wax. Images to assess the atherosclerotic lesion area and total area of the aorta were taken with a macroscopic CCD camera and analysed by computer-assisted image analysis (NIS-Elements software, Nikon Instruments, United Kingdom).

### Histological staining of the aortic root

Murine aortic sinus samples were obtained by excising the heart and transecting parallel to the atria. Following fixation in 10% formalin (v/v) buffered saline for at least 24 h, samples were serially cut (at 5 μm intervals) from the valve leaflets until the beginning of the aorta. Sections where all 3 valve leaflets were visible at different stages of the plaque development were selected. To determine lesion size and plaque features—necrotic core and fibrous cap thickness—sections were deparaffinised in xylene, rehydrated and stained with Elastic stain kit (Verhoeff Van Gieson/EVG Stain—ab150667) following manufacturer’s instructions. To determine collagen content, Picrosirius Red staining (ab246832) was used to detect fibrillar collagen networks, where the intensity threshold was set up from the mean threshold across multiple images from the same plaque. Lesion area relative to lumen area was calculated at different regions of the plaque, taken as the mean per plaque. Fibrous cap thickness was measured relative to the thickness of the plaque, taken as the mean per plaque. For immunohistochemistry, consecutive cross-sections were stained to assess smooth muscle cell (SMC) and macrophage content. SMC content was assessed using mouse anti-human smooth muscle actin (Dako, M0851) that required a mouse-on-mouse (MOM) staining kit (Vector, MP-2400). VectaFluor ready-to-use DyLight dye-labelled secondary antibody kit was used for fluorescence staining (Vector, DI-2794). For macrophage content, slides were incubated with Mac-3 (BD, 550292) and with secondary antibody Goat anti- Rat DyLight 488 (ImmunoReagents).

### Bone marrow derived macrophage isolation and culture

Bone marrow was isolated from the femurs and tibias of *Trib3^KO^* and *Trib3^WT^* mice under aseptic conditions. The bone marrow was flushed using a syringe and 26-guage needle into RPMI 1640 medium + 10% (v/v) ultra-low endotoxin heat-inactivated FBS. The cell suspension was passed through a 40 μm cell strainer and centrifuged at 500 *g* for 5 min. The samples obtained could then be frozen after resuspension in 90% (v/v) ultra-low endotoxin heat inactivated FBS + 10% DMSO or cultured for further gene analysis.

### Gene expression analysis by RT-qPCR

RNA isolation from macrophage in culture was performed using the RNeasy UCP kit (Qiagen) according to manufacturer’s instructions. cDNA was transcribed from the total RNA using the Precision nanoScriptTM 2 RT kit (Primerdesign Ltd.) according to the manufacturer’s instructions. Real time qPCR was performed using a Bio-Rad i-Cycler. The reagents used were Precision PLUS SYBR-Green master mix (Primerdesign) and specific primers (Supplementary Table 1) designed with NCBI BLAST. All assays were performed in triplicate and normalised to the expression levels of GAPDH, determined in our assays as the most suitable house-keeping gene in Trib3 deficient macrophages (Supplementary Figure 1).

### Cytoskeleton staining and analysis

Bone Marrow Derived Macrophages (BMDMs) and hMDMs were fixed with 4% (v/v) paraformaldehyde (PFA) and permeabilised with 0.1% (v/v) Triton-X diluted in PBS following staining with Fluorescein Isothiocyanate Labelled Phalloidin (Sigma-Aldrich). The slides were mounted with antifade mounting medium with DAPI (Life Technology) and coverslips. All the samples were kept in the dark until imaging. Images to assess cell morphology were taken using a Leica AF6000 microscope at 10× and 20× magnification. Three fields of view per tissue per sample were captured and analysed using Image J software. The same threshold was set in all images and by using the “analyse particles” command, the above- mentioned features were selected for automated quantification analysis. Under circularity, values closer to “1” correspond to particles that have similar shapes to a perfect circle, if the value approaches zero the particle contains spindle shaped features and/or increased elongation. The aspect ratio (AR) was used to measure elongation, for this measurement the software fits an ellipse within each particle/cell and calculates the ratio within the major and minor axes, therefore an increased AR value corresponds to an increased cell elongation.

### Scanning electron microscopy

BMDMs from *Trib3^WT^* and *Trib3^KO^* mice were cultured for 5 days in complete media supplemented with L929 (M-CSF rich) and seeded in coverslips to allow adhesion. After differentiation, all samples were fixed in 4% (v/v) PFA for 10 min followed by 2.5% (v/v) glutaraldehyde in 0.1 M sodium phosphate overnight. Thereafter, samples were washed with 0.1 M phosphate buffer at least twice with 10-min intervals. A secondary fixation in 2% (v/v) aqueous osmium tetroxide was performed. Samples were then dehydrated sequentially using 75, 95, and 100% (v/v) ethanol before drying using a Leica EM CPD300. When dried, samples were mounted on aluminium stubs, attached to carbon sticky tabs, and coated with 25 nm of gold in an Edwards S150B sputter coater. Images to assess cell morphology were taken using TESCAN Vega 3 LMU Scanning Electron Microscope at an acceleration voltage of 10 kV. Cell morphology was analysed using Image J software. After setting the same threshold and smoothing the background in all images, by using the “analyse particles” command, the cell surface area and elongation (as aspect ratio) features were selected for automated quantification analysis. For membrane ridge analysis, an optimised image analysis workflow based on *ridge detection* (29) was used to estimate ridge density in single cell SEM images. The ridge area density was calculated as ridge total cross-sectional area/cell cross-sectional area.

### Statistical analysis

All statistical analysis and graphs were generated in GraphPad Prism 8 software, with the average of at least three independent replicates (individual animals, human donors or patients) with standard error of the mean shown. Differences between two or more groups were assessed using two-tailed Student’s *t*-test, one- or two-way ANOVA, assuming normal distribution and considering *p*-values < 0.05 as significant.

## Results

### Upregulation of tribbles 3 in unstable regions of human carotid artery plaques

**FIGURE 1:**
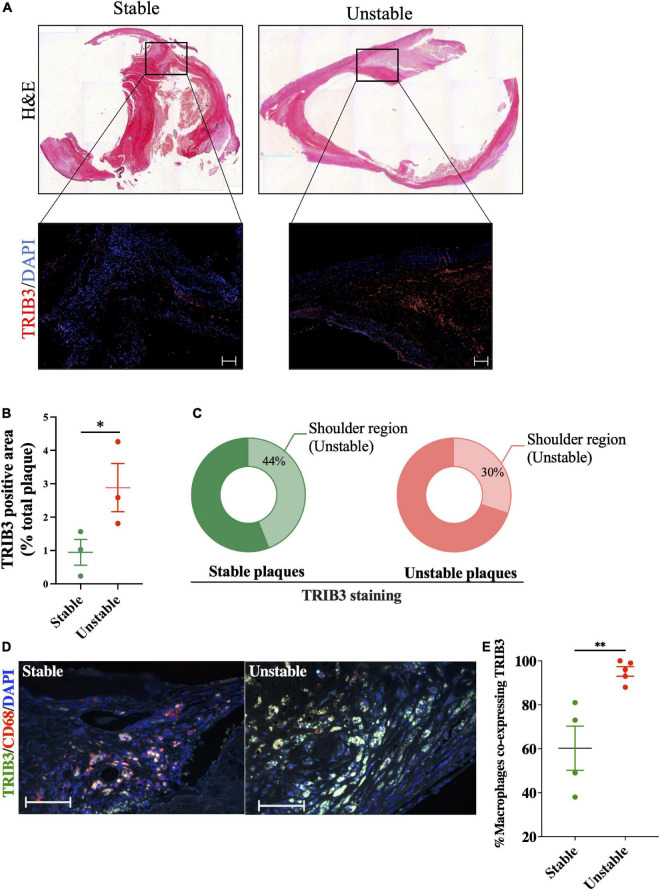
TRIB3 expression is increased in human unstable carotid plaques, in unstable plaque regions and in macrophages from unstable plaques, relative to stable plaques. **(A)** H&E staining of human carotid plaques was used to identify plaque features, classified as stable or unstable using the Stary classification guidelines. Below, representative images of the shoulder region of these plaques with TRIB3 staining (red) with DAPI (blue) as a nuclear marker (scale bars represent 100 μm). **(B)** Quantification of TRIB3 expression, as a percentage of the plaque area, of stable (0.944 ± 0.38) and unstable (2.88 ± 0.72) plaques. **(C)** Percentage of the total TRIB3 staining in the shoulder region (stable: 43.71 ± 9%; Unstable: 56 ± 9%). **(D)** Representative images of TRIB3 and CD68 macrophage co-staining from stable and unstable plaques (scale bars represent 100 μm). **(E)** Quantification of percentage CD68 + macrophages co-expressing TRIB3 in stable and unstable plaques. Data is shown as the mean ± SEM; each data point represents one donor (*N* = 3–5 patients). Unpaired *t*-test, *p < 0.05, ^**^p < 0.01.

Tissue sections from carotid plaques from human patients undergoing endarterectomy, stained with H&E were classified based on the Stary classification (30) to determine their stability (Figure 1A). Sequential sections from these carotid plaques were used to assess expression of TRIB3 according to disease severity and regions associated with atherosclerotic plaque instability (Figure 1A). Quantification of TRIB3 positive areas showed an increased expression in unstable plaques (Figure 1B). Staining of TRIB3 showed it was not widely expressed within the whole plaque, but was found predominated in the shoulder region, considered vulnerable sites for plaque rupture. The percentage of total TRIB3 + ve shoulder regions of stable plaques represented 44% of the total signal (Figure 1C), suggesting that TRIB3 is upregulated in unstable areas. In unstable plaques 30% of TRIB3 staining was found in this region while the remaining staining was expressed diffusely across other areas. Double staining of carotid plaques for TRIB3 and the pan-macrophage marker, CD68, demonstrated that unstable plaques show an increased proportion of macrophages with high expression of TRIB3, compared to stable plaques (Figures 1D,E). Overall, these initial results suggest that TRIB3 is upregulated in unstable plaque macrophages and is more highly expressed in the unstable (shoulder) regions of atherosclerotic lesions compared to stable regions, raising the possibility that macrophage TRIB3 may play a regulatory role in plaque stability.

### Atherogenic diet-fed *Trib3^KO^* mice have increased body mass and expanded visceral adipose

**FIGURE 2:**
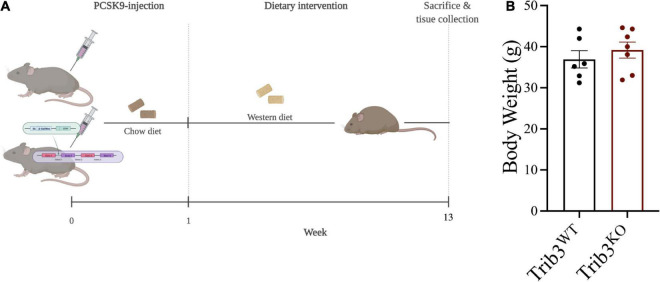
Atherosclerosis model and weight gain in *Trib3^KO^* mice. **(A)** Schematic representation of atherosclerosis development model used in Trib3*^WT^* and Trib3*^KO^* mice. **(B)** Body weight measurements in Trib3*^WT^* and Trib3*^KO^* mice at 13 weeks.

Since we found that TRIB3 was upregulated in vulnerable and unstable regions of human plaques we used a *Trib3^KO^* mouse model of atherosclerosis to assess the consequences of Trib3-deficiency in atherosclerosis development and in fibrous cap formation. Twelve week-old male *Trib3^KO^* and *Trib3^WT^* mice were injected with adeno-associated-virus-8, over-expressing the LDLR inhibitor PCSK9 (rAAV8-PCSK9). One week after injection, mice were fed with Western diet for 12 weeks (Figure 2A). Body weight was monitored weekly (Figure 2B) with no overall difference in weight gain between *Trib3^KO^* and *Trib3^WT^* mice, although *Trib3^KO^* started and maintained a higher average weight during the experiment (Supplementary Figures 2A,B).

Analysis of the visceral adipose tissue, liver and spleen mass showed a significant increase in adipose tissue expansion in *Trib3^KO^* mice with no differences in liver or spleen weights (Supplementary Figure 2C). Despite an increased body weight and adiposity in *Trib3^KO^* mice, there were no differences in levels of circulating triglycerides, cholesterol (total, HDL, and non-HDL), and glucose (Supplementary Figure 2D).

### Reduced atherosclerotic burden and increased plaque cap thickness in *Trib3^KO^* mice

**FIGURE 3:**
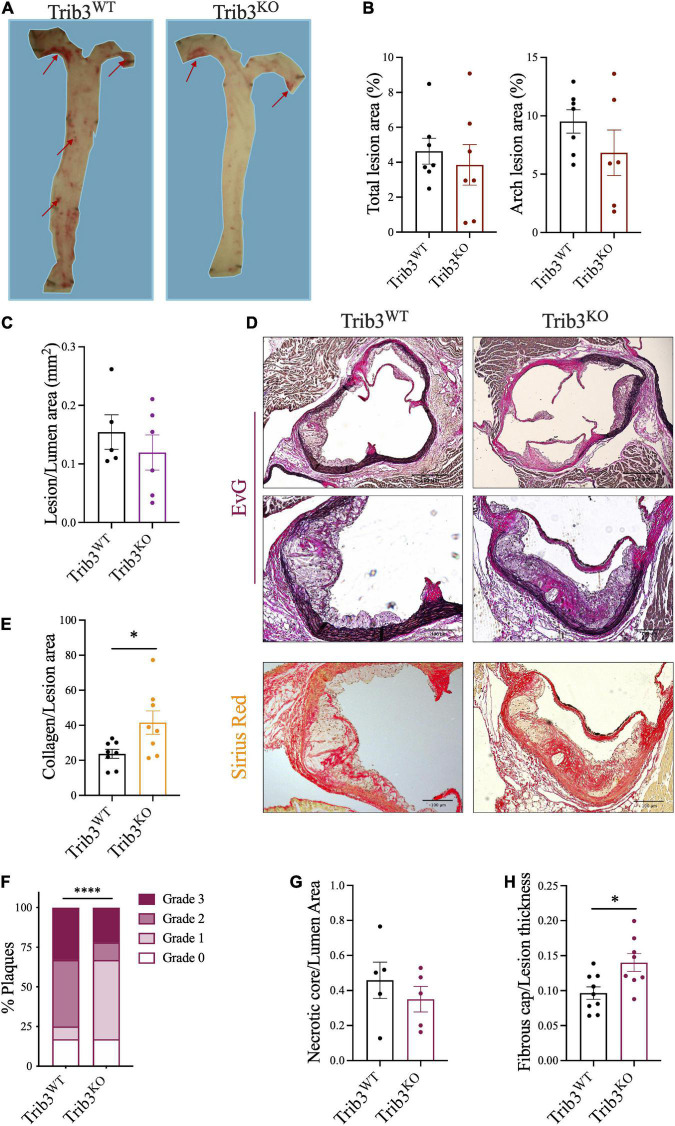
Atherosclerotic lesion size, disease progression and features in Trib3*^KO^* mice. **(A)** Representative images and quantification **(B)** of atherosclerotic lesions stained with ORO in the inner lumen of the aorta and in the aortic arch. **(C)** Total lesion quantification in the aortic root. Graphs are shown as the mean ± SEM, each data point represents a single mouse, unpaired *t*-test. **(D)** Representative images of Elastic van Gieson [elastin, original, and inset (high power image)] and picosirius red (collagen) stained cross-sections of aortic root lesions. **(E)** Collagen content quantification using picrosirius staining shown as mean ± SEM where each data point represents a single lesion; un-paired *t*-test *p < 0.05. **(F)** Double-blind plaque grading, made according to classification in Supplementary Table 2, presented as percentage of the total number of lesions (*n* = 14–17 lesions) using a Chi-square test, ^****^p < 0.0001. The necrotic core relative to lumen area **(G)** and fibrous cap relative to lumen thickness **(H)** quantification shown as mean ± SEM where each data point represents a single lesion where more than one lesion was assessed per individual mouse for a *N* = 6 mice for each group; un-paired *t*-test *p < 0.05. Scale bars represent 100 μm.

Analysis of lipid deposition by Oil Red O staining of the aorta and in the aortic arch (inner curvature and carotid bifurcations) in *Trib3^WT^* and *Trib3^KO^* mice showed that mean atherosclerotic burden did not differ between genotypes (Figures 3A,B). Lesion size within the aortic roots was unaltered between *Trib3^WT^* and *Trib3^KO^* genotypes (Figure 3C). Elastin and collagen content in the aortic root lesions was assessed using Elastic van Gieson (EvG) and picosirius red staining (Figure 3D), respectively, where collagen content was increased in *Trib3^KO^* plaques (Figure 3E). Atherosclerosis development and lesion character in *Trib3^WT^* and *Trib3^KO^* mice was assessed by grading individual plaques in relation to the stage of the disease as summarised in Supplementary Table 2 and described in Otsuka et al. (31). Initial lesions with adaptive intimal thickening consisting mainly of smooth muscle cells and isolated macrophage foam cells were classified as Grade 0; Intimal xanthoma or “Fatty steak” lesions were characterised by the accumulation of macrophages within the intima and intracellular lipid accumulation and classified as Grade 1. Grades 0 and 1 are considered early manifestations of the disease and the precursors of symptomatic atherosclerosis. Grade 2 were plaques showing pathological intimal thickening, characterised by the presence of lipid pools or small necrotic cores with or without thinner fibrous caps consisting of SMC. Fibroatheroma lesions were classified as Grade 3, these plaques contained larger necrotic cores characterised by increased macrophage apoptosis and encapsulated by fibrotic tissue and collagen forming the fibrous cap.

In a double-blinded analysis, all plaques were graded following these guidelines, revealing that *Trib3^KO^* mice present with less advanced disease with an increased content characteristic of early plaques (17% grade 0; 50% grade 1) and fewer advanced plaques (11% grade 2; 22% grade 3, Figure 3F). Wild-type littermates had a greater number of advanced lesions (42% grade 2; 33% grade 3) and fewer early plaques (17% grade 0; 8% grade 1, Figure 3F).

The association of plaque structure with plaque stability was further assessed by quantification of necrotic core area and fibrous cap thickness. No significant changes were detected in necrotic core areas (Figure 3G), however, plaques from *Trib3^KO^* mice had a significant increase in fibrous cap thickness (Figure 3H). These data, taken together with the twofold increase in collagen in plaques (Figure 3E) suggest that the absence of *Trib3* does not affect atherosclerotic lesion size *per se*, but that it reduces the pathobiological complexity of early atherosclerotic disease associated with increased plaque stability in more advanced lesions.

### Increased macrophage content in *Trib3^KO^* atherosclerotic plaques

**FIGURE 4:**
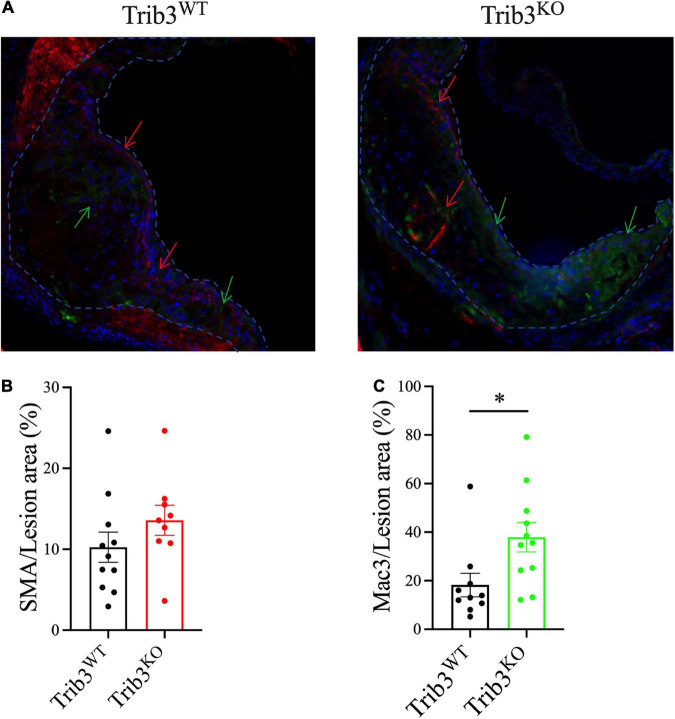
Genetic deletion of Trib3 has no effect on SMC content but increases macrophage presence in advanced atherosclerotic lesions with altered ECM degradative function. **(A)** Representative images of α-SMA (red) and MAC-3 (green) staining of aortic root lesions from Trib3^KO^ and Trib3^WT^ mice. DAPI (blue) was used as a marker for nuclear staining. Quantification of the SMC area **(B)** and macrophage area **(C)** immunostaining as a percentage of the total lesion area. Graphs are shown as the mean ± SEM; each data point represents a lesion (*n* = 9–10 lesions from *N* = 6 mice for each group). Unpaired *t*-test, *p < 0.05.

Since *Trib3* deletion increased features associated with plaque stability, mouse lesions were analysed for changes in smooth muscle cell and macrophage cell content, as major contributors to the development of stable vs. unstable plaque phenotypes. Sequential cross-sections of aortic root lesions from *Trib3^KO^* and *Trib3WT* mice were stained with α-SMA and MAC-3, to quantify the levels of SMC and macrophage content in the plaques, respectively (Figure 4A). Although *Trib3^KO^* plaques have increased fibrous caps, image analysis showed that smooth muscle cell content did not differ between *Trib3^KO^* and *Trib3WT* genotypes (Figure 4B). While an increase in macrophage content has traditionally been associated with reduced plaque stability (32) quantification of MAC3-positive staining showed a significant increase in plaque macrophages from *Trib3^KO^* mice (Figure 4C), suggesting that increased macrophage content, rather than smooth muscle cell content alone, may be driving the increase in fibrous cap thickness observed in *Trib3^KO^* lesions. To elucidate the molecular mechanisms regulating fibrous cap thickness, we next sought to analyse the consequences of *Trib3* deficiency on the macrophage phenotype.

### Loss of tribbles 3 dysregulates extracellular matrix remodelling pathways in human and mouse macrophages

**FIGURE 5:**
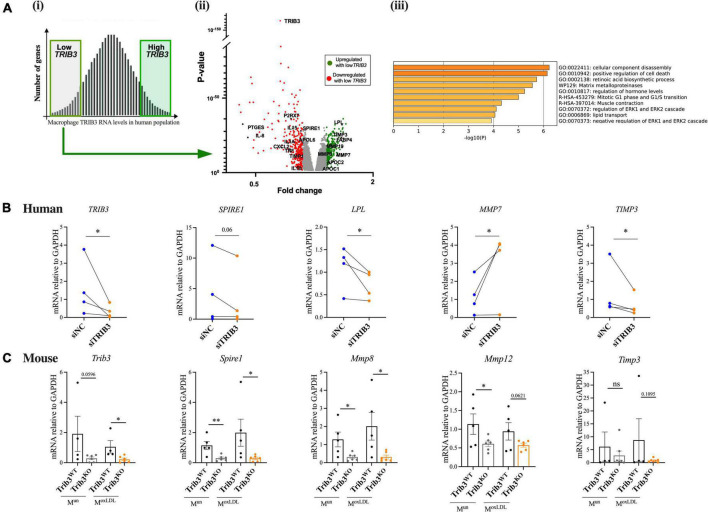
Loss of TRIB3 alters extracellular matrix remodelling pathways in human and mouse macrophages. **(Ai)** Microarray data of MDMs from 596 human donors was ranked based on *TRIB3* expression and the top high and bottom low expressors were selected for further analysis. **(ii)** Under a low TRIB3 expression condition, the volcano plot shows the significant genes correlatively expressed with TRIB3, passing the cut off *p*-value < 0.05 and passing threshold fold change < 0.8 (red—downregulated, so correlatively expressed, with low *TRIB3* expression; examples include *P2* × *7, PTGES, IL-8, IL15, IL1A, SPIRE1, APOL6, SXSL2, TNF, TIMP1, IL1B*) and > 1.2 (green—upregulated, so inversely correlated, with low TRIB3 expression; examples include *LPL, TIMP3, FABP4, MMP19, MMP8, MMP7, APOC2, APOC1*). **(iii)** Functional gene set enrichment analysis using Metascape for genes inversely correlated with low *TRIB3* expression. **(B)** si*TRIB3*-knockdown mediated changes in mRNA expression levels of genes involved in lipid metabolism (LPL), cytoskeleton and ECM remodelling (*SPIRE1*, *MMP7*, and *TIMP3*) in human MDMs. Graphs are presented as individual connected points representing one donor, paired student’s *T*-test, *p < 0.05. **(C)** Expression of mouse *Trib3* and ECM remodelling genes (*Spire1, Mmp8, Mmp12, and Timp3*) in *Trib3^WT^* and *Trib3^KO^* BMDMs treated or untreated with oxLDL. Graphs are shown as the mean ± SEM (*N* = 4–6). Unpaired *t*-test, *p < 0.05, ^**^p < 0.01. GAPDH expression was used as an internal control.

First, we explored whether the levels of *Trib3* RNA levels in macrophages are associated with an altered regulation of genes associated with increased plaque stability. To achieve this, we analysed the gene expression profile of human primary monocyte-derived macrophages (MDMs) from the Cardiogenics Consortium transcriptomic data set (21–23) of 596 donors. Samples were ranked based on *TRIB3* expression then transcriptomic profiles between the upper and lower quartiles were compared. Differentially expressed genes between the samples with high and low *TRIB3* levels were identified. A total of 5,782 significant genes were found to be differentially expressed between high vs. low *TRIB3* expression. Genes were identified that were correlatively expressed for low *TRIB3* (fold change = 0.32 from the mean); these were classified as directly correlated (fold change < 0.8) vs. inversely correlated (fold change > 1.2) to *TRIB3* expression, respectively (Figure 5A and Supplementary Data).

Analysis of the genes in the Cardiogenics dataset that significantly correlated with low human MDM *TRIB3* expression were entered into a functional gene set enrichment analysis using the DAVID Functional Annotation Analysis Software and Metascape. As summarised in Table 1 and Figure 5Aiii lower levels of *TRIB3* in macrophages were significantly associated with metabolic, cardiovascular, and pharmacogenomic diseases. In the cellular component analysis, altered genes associated with *TRIB3* downregulation were significantly enriched for “extracellular exosome,” “Cytosol,” “Cytoplasm,” “Plasma membrane,” “Extracellular space” and “Extracellular region.” In the analysis of biological functions, the inflammatory response was most significantly enriched (FDR = 2.2 × 10^6^). Extracellular matrix disassembly was altered (FDR = 7.9 × 10^2^; *p* = 2.0 × 10^4^), notably with matrix regulatory genes *MMP7, LPL, TIMP3, MMP8, COL22A1, MMP19, SPIRE1*, and *TIMP1* in the top 5% of the 5,782 genes significantly altered between high vs. low *TRIB3* expression (Figure 5Aii).

**TABLE 1:** Functional gene ontology analysis of human macrophages with low *TRIB3* expression.

Disease	Gene count	*P*-value	FDR
Metabolic	195	2.2 × 10^–3^	5 × 10^–3^
Cardiovascular	154	2.6 × 10^–3^	4.2 × 10^–2^
Chem dependency	143	1.5 × 10^–3^	4.4 × 10^–3^
Cancer	140	9.1 × 10^–6^	5.5 × 10^–5^
Pharmacogenomic	124	5.6 × 10^–6^	5 × 10^–5^
Neurological	111	1.4 × 10^–2^	2.4 × 10^–2^
Immune	111	3.3 × 10^–2^	4.9 × 10^–2^
**Biological process**			
Inflammatory response		9 × 10^–10^	2.2 × 10^–6^
tRNA aminoacylation		9.5 × 10^–6^	1.2 × 10^–2^
Amino acid transport		3.5 × 10^–5^	2.8 × 10^–2^
Chemotaxis		1.1 × 10^–4^	6.8 × 10^–2^
Response to LPS		1.5 × 10^–4^	7.0 × 10^–2^
Extracellular matrix disassembly		2.0 × 10^–4^	7.9 × 10^–2^
Prostaglandin metabolic process		3.1 × 10^–4^	1 × 10^–1^
**Cellular component**			
Extracellular exosome		9.4 × 10^–8^	3.3 × 10^–5^
Cytosol		2.7 × 10^–7^	4.7 × 10^–5^
Cytoplasm		5.5 × 10^–5^	6.4 × 10^–3^
Plasma membrane		6.5 × 10^–5^	5.7 × 10^–3^
Extracellular space		1.5 × 10^–4^	1.0 × 10^–2^
Extracellular region		6.0 × 10^–4^	3.5 × 10^–2^
Integral component of plasma membrane		2.5 × 10^–3^	1.2 × 10^–1^

Significantly altered gene expression correlating with low human MDM TRIB3 expression were analysed from the Cardiogenics dataset by functional gene set enrichment analysis using the DAVID Functional Annotation Analysis Software.

To substantiate whether these *TRIB3*-correlative transcriptomic changes in macrophages showed possible causality, expression of the most significant and altered genes associated with low *TRIB3* expression in the human microarray was assessed in human MDMs transfected with *TRIB3*-targeting siRNA, compared to non-targeting siRNA. The efficiency of *TRIB3* gene silencing was 46 ± 9%, determined by quantification of *TRIB3* mRNA levels (Figure 5B). The effect of *TRIB3* knockdown on expression of selected extracellular matrix remodelling genes was evaluated (Figure 5B). In some cases, changes in the opposite direction were detected in the analysis of gene expression by qRT-PCR compared to the microarray data, likely reflecting differences between *TRIB3* knockdown compared to correlative, co-expression analysis of the Cardiogenics data. *TRIB3* knockdown resulted in significant downregulation of *LPL* (lipoprotein lipase) expression, a driver of lipid metabolism and lipoprotein uptake. A significant reduction in expression of *TIMP3*, a metalloproteinase inhibitor, was also seen in the *TRIB3* knockdowns (Figure 5B). Conversely, *TRIB3* knockdown led to increased expression of matrix metalloproteinase *MMP7* in contrast to the lower expression seen in the Cardiogenics dataset in relation to TRIB3 expression.

Key genes in the differentially expressed human macrophage extracellular matrix remodelling pathways identified in the Cardiogenics dataset were then compared in bone marrow derived macrophages (BMDMs) isolated from *Trib3WT* and *Trib3^KO^* mice. BMDMs from *Trib3WT* and *Trib3^KO^* mice were treated with oxLDL to mimic the plaque foam cell phenotype. *Trib3* expression in these cells confirmed the genotype of the *Trib3WT* and *Trib3^KO^* samples (Figure 5C). *Spire1*, an actin nucleation factor involved in the actin cytoskeleton organisation and actin nucleation whose alteration affects cell shape and ECM remodelling, was downregulated in both unpolarised and oxLDL-treated *Trib3^KO^* macrophages. *SPIRE1* expression was also compared in human MDMs where a decreased expression with TRIB3 knockdown was observed in 2 out of 3 donors tested (Figure 5C). *Timp3* regulates MMPs and therefore plays a key role in extracellular matrix composition; its mRNA levels were not significantly altered upon oxLDL-treatment or *Trib3* downregulation. Relative expression levels of the collagenolytic *Mmp8* and elastinolytic *Mmp12* metalloproteinases that degrade components of the ECM were also significantly reduced in *Trib3^KO^* unpolarised and oxLDL-treated macrophages. Taken together, these data indicate that *Trib3* regulates extracellular matrix remodelling by reducing metalloproteinase-8 and −12 expression in foamy macrophages, accounting for the increased collagen and fibrous cap thickness seen in by *Trib3^KO^* atherosclerotic lesions.

### Loss of tribbles 3 results in cellular elongation, increased surface area, increased actin, and altered polarisation of macrophages

**FIGURE 6:**
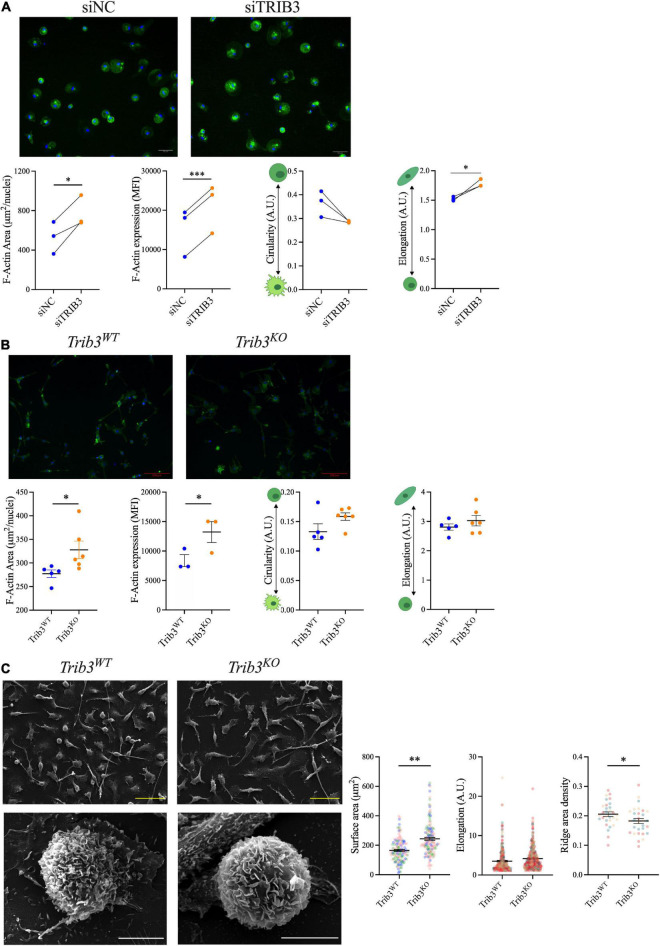
Increased F-actin and size of macrophages with reduced TRIB3 expression. **(A)** F-actin staining and quantification of human MDMs with non-targeting or siTRIB3-knockdown; representative images of phalloidin (green) and Dapi (blue) staining (upper) with quantification of cell size (F-actin area), F-actin mean fluorescence intensity and shape descriptors (circularity and elongation), shown connected points for each donor, compared by paired student’s *T*-test, *p < 0.05, ^***^p < 0.001. **(B)** F-actin staining and quantification of Trib3*^WT^* and Trib3*^KO^* BMDMs; representative images of phalloidin (green) and Dapi (blue) staining (upper) with quantification of cell size (F-actin area), F-actin mean fluorescence intensity and shape descriptors (circularity and elongation). Shown as Mean ± SEM where each data point is the average of all cell measurements per individual mouse (3 fields of view per mouse *N* = 3–6) unpaired student’s *t*-test, *p < 0.05 **(C)** Trib3*^WT^* and Trib3*^KO^* BMDMs imaged by scanning EM; quantification of cell surface area, elongation and ridge area density shown as Mean ± SEM where each data point is from an individual cell (3 fields of view per mouse *N* = 3, 30–60 cells for surface area; 95–148 cells for elongation; 10 cells for ridge density measurements) compared by unpaired student’s *T*-test, *p < 0.05, ^**^p < 0.01. Scale bars represent 100 μm.

Since *TRIB3* knockdown or knockout in both human and mouse macrophages alters the expression of genes in extracellular matrix pathways, we assessed the functional impact of reduced *TRIB3* levels on macrophage cell morphology and cytoskeleton. Human MDMs transfected with *TRIB3*-targeting siRNA, compared to non-targeting siRNA, were stained with F-actin-binding fluorescein isothiocyanate labelled-phalloidin. Cell size, F-actin intensity and shape descriptors (circularity and elongation) were analysed by fluorescence microscopy. This showed that *TRIB3* downregulation led to an increase in cell size, measured as F-actin area per nuclei, as well as increased actin expression, measured as MFI (Figure 6A). Morphological analysis of *siTRIB3* macrophages showed no significant changes in cell circularity, while a significant increase in elongation was detected compared to non-targeting siRNA control transfected cells (Figure 6A).

**FIGURE 7:**
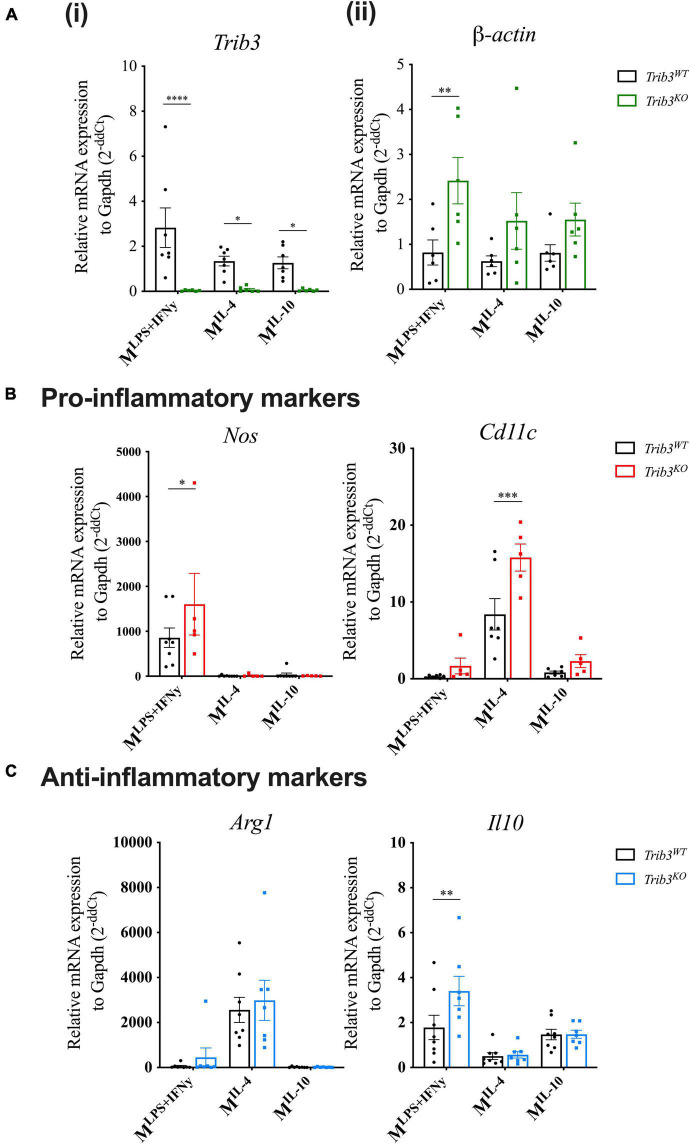
*Trib3^KO^* BMDMs show altered gene expression after polarisation. qPCR gene expression analysis after macrophage polarisation shows changes in the expression levels of **(Ai)** *Trib3* to confirm the knockout genotype, **(ii)** β*-actin* for changes in the cytoskeleton, **(B)** and pro-inflammatory markers (*Nos2* and *Cd11c)* and **(C)** anti-inflammatory markers *Arg1* and *Il*-*10*. *Gapdh* expression was used as an internal control and normalised to unpolarised controls to give 2^–ΔΔCt^ values. Graphs are presented as Mean ± SEM, two-way ANOVA with Fisher’s *post hoc* comparisons, *p < 0.05, ^**^p < 0.01, ^***^p < 0.001, ^****^p < 0.0001.

To assess the cellular consequences of TRIB3-dependent changes in cytoskeleton and extracellular matrix in murine macrophages, the morphology of BMDMs from *Trib3WT* vs. *Trib3^KO^* mice was examined (Figures 6B,C). Phalloidin staining analysis showed that *Trib3^KO^* macrophages have an increased cell size (327.9 ± 18.50 μm^2^) compared to *Trib3WT* (277.4 ± 8.058 μm^2^) and a significant increase in F-actin mean fluorescence intensity (MFI) staining. In line with this, increased β-actin expression was detected by qPCR in *Trib3^KO^* BMDMs compared to WT (Figure 7A).

Changes in cellular morphology and elongation occur with macrophage polarisation. Polarised *Trib3^KO^* BMDMs showed a relative increase in inflammatory markers *Nos2*, when polarised with inflammatory cytokines (LPS + IFNγ), and *Cd11c* for polarisation with the anti-inflammatory cytokine, IL-4; while the anti-inflammatory marker *Il10* was increased in *Trib3^KO^* BMDMs polarised with inflammatory cytokines (Figures 7B,C). Unlike the human MDMs, no differences in cell circularity or elongation were detected between the genotypes (Figure 6B). However, detailed examination of the morphological features of the *Trib3WT* and *Trib3^KO^* BMDMs using scanning electron microscopy showed that *Trib3^KO^* macrophages have increased cell size without affecting cell elongation, with a significant reduction in cell membrane ridges (Figure 6C).

## Discussion

TRIB-3 modulates lipid and glucose metabolism, macrophage lipid uptake, with a gain-of-function variant associated with increased cardiovascular risk (17). In the current study, TRIB3 protein expression was increased in unstable regions of human carotid artery plaques. Using a mouse model of atherosclerosis, loss of TRIB3 resulted in increased macrophage numbers in atherosclerotic plaques and increased plaque stability features of thicker fibrous caps and an increase in collagen. Reduced or absent TRIB3 in human and mouse macrophages, respectively, led to altered expression of extracellular matrix metalloproteinases and actin remodelling pathways. This also led to an increased macrophage actin expression, increased cellular size and elongation suggesting a more reparative phenotype (33). Together these results suggest TRIB3 upregulates macrophage matrix production and reduces MMP expression, resulting in weakened fibrous caps and potentially increased plaque instability associated with acute cardiovascular disease events.

TRIB3 has been proposed as a key target to treat obesity-related diseases, as many studies have shown that its overexpression prevents the detrimental effects of diet-induced obesity by modulating lipid metabolism (9), promoting insulin resistance (10–12) and inhibiting adipocyte differentiation (13–15). In addition, in the context of inflammation and cardiovascular disease, loss of TRIB3 has been implicated in the development of diabetic atherosclerosis ameliorating insulin resistance and reducing macrophage apoptosis *via* modulating AKT phosphorylation (34, 35). TRIB3 upregulation has been associated with the development of a lean phenotype (9) and its over-expression in macrophages has been shown to promote cholesterol accumulation and reduced expression of pro-inflammatory cytokines (20).

Whilst previous studies have focused on the well-established roles of TRIB3 as a metabolic and AKT modulator, this is the first study to show *in vivo* and *in vitro*, using both human and murine models, that TRIB3 is mechanistically involved in modulating mechanisms associated with plaque stability. Here we have shown a clear relationship between the levels of TRIB3 and plaque stability in human carotid atherosclerotic plaques, with significantly upregulated expression in unstable compared to stable plaques. TRIB3 staining was found to be highly concentrated in macrophages within the shoulder region, considered unstable vulnerable sites of rupture where pro-inflammatory macrophages are predominantly localised (36).

Here we have shown that genetic deletion of *Trib3* mediates changes in body weight supporting effects for atherosclerosis development *in vivo*. Along with an increased body weight, *Trib3^KO^* mice also presented an increased adiposity. There were, however, no changes in plasma lipid levels compared to WT littermates, likely to be a result of increased lipid tissue storage. The similar atherosclerotic lesion coverage in both aorta and aortic roots of *Trib3WT* and *Trib3^KO^* mice may reflect the similar levels of circulating lipids in both mouse genotypes. This atherosclerotic model for *Trib3WT* and *Trib3^KO^* mice showed no differences in blood glucose contrasting with an association of TRIB3 overexpression in human insulin resistance (11) and a diabetic mouse *Trib3* siRNA knockdown, likely reflecting the difference in models and upregulation in disease. Changes in the atherosclerotic plaque morphology were noticeable visually, therefore a double blinded plaque classification analysis was carried out, revealing that *Trib3^KO^* mice had an increased number of early lesions, while *Trib3WT* mice had more advanced fibroatheromas. Although the lesion size was not affected by genetic deletion of *Trib3*, plaque character was altered with plaques from *Trib3^KO^* mice having significantly thicker fibrous caps and increased collagen content, indicating stable atherosclerotic disease.

Our results fit with other findings using a diabetic atherosclerosis model, combined with siRNA downregulated *Trib3*, where decreased atherosclerosis development with increased plaque stability features was observed with reduced macrophage apoptosis and increased phagocytosis suggested to prevent expansion of the necrotic core (18). Our study on whole body genetic deletion of *Trib3* in a non-diabetic model of atherosclerosis also promotes mechanisms associated with increased plaque stability, by a reduction in the advancement of the plaque, with increased collagen and fibrous cap thickness, thereby delineating the impact of systemic/metabolic challenges from local changes in plaque-resident cell phenotype and function. We studied the cellular composition of the plaques since the formation of the fibrous cap is a multifactorial process with vascular smooth muscle cells (VSMC) and macrophages playing key roles in balancing the production and degradation of collagen, that ultimately determines the cap strength (37). VSMCs are the major collagen source in the artery wall; while their proliferation and migration promote plaque stabilisation, an increased production of inflammatory cytokines by activated immune cells will induce apoptosis and inhibit collagen synthesis in VSMC (38). In this regard, plaques from *Trib3^KO^* mice did not exhibit a difference in VSMC content. We cannot completely rule out a contribution of TRIB3 to VSMC phenotypes in atherosclerosis; future use of conditional Trib3-knockout mouse strains for myeloid and VSMC specific expression, will help us understand these specific cellular contributions to by Trib3 to plaque stability, VMSC phenotype and the type of collagen production, beyond the scope of this current study.

An increased macrophage content is conventionally considered a sign of plaque instability, as are inflammatory macrophage phenotypes. Mouse macrophages deficient in *Trib3* showed altered expression of markers of polarisation following treatment with pro-inflammatory or anti-inflammatory cytokines where both inflammatory (“M1”) and anti-inflammatory (“M2”) markers were upregulated. Upregulation of these markers was polarisation treatment-dependent appearing more complex than simple phenotype switching. Interestingly others have found that overexpression of TRIB3 in human THP-1 macrophages has been shown to increase lipid accumulation and foam cell formation, with a reduction in inflammatory TNF secretion (20). Despite increased macrophage numbers being viewed as a sign of plaque instability, in contrast *Trib3^KO^* plaques had increased fibrous cap thickness and features of stability. Macrophages are one of the main sources of matrix metalloproteinases within the atherosclerotic plaque (39). Since we found increased fibrous cap thickness and macrophage content in *Trib3^KO^* lesions we sought to determine whether *Trib3^KO^* macrophages show an altered regulation of extracellular matrix associated with increased plaque stability.

We assessed mechanisms that could explain the role of TRIB3 in modulating gene expression and plaque macrophage phenotype using the Cardiogenics Consortium human macrophage transcriptomic dataset. When assessing causality, significant differences for macrophage TRIB3 downregulation were associated with genes involved in extracellular matrix remodelling and cell morphology regulation, with enrichment in cellular component annotations for the cytoskeleton and extracellular regions. This suggests *Trib3* activation may reduce fibrous cap thickness and promote plaque instability, through macrophage-mediated extracellular matrix destruction. We assessed this *in vitro* in *Trib3^WT^* and *Trib3^KO^* BMDMs treated with oxLDL, to promote foam cell formation and mimic the atherosclerotic plaque microenvironment. The absence of *Trib3* resulted in reduced expression of *Spire1*, a regulator of extracellular matrix degradation and MMP vesicular trafficking (40). In addition, *Mmp8* and *Mmp12* levels were reduced in *Trib3^KO^* macrophages, whose depletion in human diseased arteries and animal models of atherosclerosis results in reduced plaque size and increased collagen content, therefore improving plaque stability (41–44).

Morphological changes in macrophages mediated by TRIB3 were assessed by F-actin immunostaining. In humans and mice, *TRIB3* silencing led not only to an increased cell size but also to a significant increase in cell elongation. Similarly, *Trib3* deletion markedly increased macrophage cell size in unpolarised murine macrophages from the *Trib3^KO^* mice, which was associated with increased actin expression levels. The effects of *Trib3* deletion on macrophage circularity and elongation were less pronounced in murine macrophages. A more detailed analysis using scanning electron microscopy of *Trib3^KO^* macrophages showed an increased cell size and a reduction of the number of cell membrane ridges in macrophages lacking *Trib3* compared to WT. These changes in macrophage ridges may link to the increased phagocytosis response previously described in Trib3 knockdown macrophages (18).

In summary, our findings suggest that TRIB3 regulates macrophage cell shape, polarity, extracellular matrix remodelling and collagen, which leads to fibrous cap thinning and a reduction in features of plaque stability. TRIB3-activating therapies to promote a healthy obese phenotype may need to be balanced with the potential to cause atherosclerotic plaque instability and risk of acute cardiovascular events.

## Data availability statement

The original contributions presented in this study are included in the article/Supplementary material, further inquiries can be directed to the corresponding author/s.

## Ethics statement

The studies involving human participants were reviewed and approved by the Human tissue and blood samples from volunteers and patients who gave informed written consent, were collected under protocols approved by the University of Sheffield Research Ethics Committee and Sheffield Teaching Hospitals Trust Review Board (ref. STH18222 and SMBRER310), Sheffield, United Kingdom, and in accordance with the Declaration of Helsinki. The patients/participants provided their written informed consent to participate in this study. The animal study was reviewed and approved by the University of Sheffield Project Review Committee approved all animal experiments which were carried out under the United Kingdom Home Office Project Licence P5395C858.

## Author contributions

HW, EK-T, and LM-C conceived and designed the experiments. LM-C, JJ, KK, CM, and JC performed the experiments. LM-C, SF, JR, KK, AG, and SH analysed the data. LM-C and HW wrote the manuscript. JJ, KK, JC, SF, AG, and EK-T contributed to the writing of the article. All authors approved the submitted version.

## Footnotes

1
https://david.ncifcrf.gov/
