# Supplementary material for: Tribbles 3 deficiency promotes atherosclerotic fibrous cap thickening and macrophage-mediated extracellular matrix remodelling
Source: Front Cardiovasc Med. 2022 Aug 26;9:948461. doi: 10.3389/fcvm.2022.948461 (PMC9505024; doi:10.3389/fcvm.2022.948461)
Supplement: Supplementary file 1 [file Data_Sheet_1.docx]

**Supplementary Information**

**Supplementary Table 1. Primer sequences used in RT-qPCR**

|  | Gene | Forward | Reverse |
| --- | --- | --- | --- |
| Human | *GAPDH* | ATTGCCCTCAACGACCACTTT | CCCTGTTGCTGTAGCCAAATTC |
|  | *TRIB3* | TGGTACCCAGCTCCTCTACG | GACAAAGCGACACAGCTTGA |
|  | *LPL* |  |  |
|  | *SPIRE1* | GGAAGCTGACGGTAGCAATGA | TGCACGACATACTGCCTGATAA |
|  | *MMP7* | GAGTGAGCTACAGTGGGAACA | CTATGACGCGGGAGTTTAACAT |
|  | *TIMP3* | CATGTGCAGTACATCCATACGG | CATCATAGACGCGACCTGTCA |
| Mouse | *Gapdh* | TGGCAAAGTGGAG ATTGTTGCC | AAGATGGTGATGGGCTTCCCG |
|  | *Trib3* | TCGACTGGGGCCTTATATCCTT | CAGGTGTACTCTGTGCCTGTG |
|  | *Lpl* | TTGCAGAGAGAGGACTCGGA | GTTGCACCTGTATGCCTTGC |
|  | *Spire1* | GCCCGGTTCTGGGTACAAG | AAGGGGTCAGTTGGTACTCAA |
|  | *β-actin* | GGGACCTGACAGACTACCTCATG | GTCACGCACGATTTCCCTCTCAGC |
|  | *Nos* | GCAGTCTTTTCCTATGGGG | TGGAACTCTGGGCTGTCAGA |
|  | *Cd11c* | TGTGTTTGAGTGTCAGGAGCA | GTCACCTAGTTGGGTCTTGGG |
|  | *Arg1* | ACATTGGCTTGCGAGACGTA | ATCACCTTGCCAATCCCCAG |
|  | *Il10* | AAGGGTTACTTGGGTTGCCA | CCTGGGGCATCACTTCTACC |
|  | *Mmp8* | CAACATTGCTTTCGTCTCAAGAG | GCATGGGCAAGGATTCCATT |
|  | *Mmp12* | CTGCTCCCATGAATGACAGTG | AGTTGCTTCTAGCCCAAAGAAC |
|  | *Timp3* | CACATCAAGGTGCCATTCAGGTAG | GTTCTCTCCTCCTCAACCCAAACA |

**Supplementary Table 2. Atherosclerotic plaque grading classification.**
