# Supplementary material for: Tribbles 3 deficiency promotes atherosclerotic fibrous cap thickening and macrophage-mediated extracellular matrix remodelling
Source: Front Cardiovasc Med. 2022 Aug 26;9:948461. doi: 10.3389/fcvm.2022.948461 (PMC9505024; doi:10.3389/fcvm.2022.948461)
Supplement: Supplementary file 2 [file Data_Sheet_2.docx]

**Supplementary Figures**
